# Connectivity reveals homology between the visual systems of the human and macaque brains

**DOI:** 10.3389/fnins.2023.1207340

**Published:** 2023-07-05

**Authors:** Xia Lu, Qianshan Wang, Xiaowen Li, Guolan Wang, Yifei Chen, Xueqi Li, Haifang Li

**Affiliations:** ^1^College of Computer Science and Technology, Taiyuan University of Technology, Taiyuan, China; ^2^Shanxi Technology and Business College, Taiyuan, China

**Keywords:** cross-species comparison, visual system, DTI, RS-fMRI, homology

## Abstract

The visual systems of humans and nonhuman primates share many similarities in both anatomical and functional organization. Understanding the homology and differences between the two systems can provide important insights into the neural basis of visual perception and cognition. This research aims to investigate the homology between human and macaque visual systems based on connectivity, using diffusion tensor imaging and resting-state functional magnetic resonance imaging to construct structural and functional connectivity fingerprints of the visual systems in humans and macaques, and quantitatively analyze the connectivity patterns. By integrating multimodal magnetic resonance imaging, this research explored the homology and differences between the two systems. The results showed that 9 brain regions in the macaque visual system formed highly homologous mapping relationships with 11 brain regions in the human visual system, and the related brain regions between the two species showed highly structure homologous, with their functional organization being essentially conserved across species. Finally, this research generated a homology information map of the visual system for humans and macaques, providing a new perspective for subsequent cross-species analysis.

## 1. Introduction

The macaque serves as a natural transitional model for studying humans and has a unique resource advantage. Exploring the working and pathological mechanisms of the human brain through macaques is an important means of brain research (Xia et al., 2021). However, the similarity and dissimilarity between the human and macaque brains have become a research hotspot and difficulty. In recent years, the accumulation and public availability of human and macaque brain imaging data have provided support for direct comparison between the two (Milham et al., 2018). Cross-species comparison methods based on imaging features can provide a unique interpretation of brain tissue organization that differs from single-species studies, and can support the discovery and hypotheses about the human brain.

The visual cortex of primates is highly developed, with approximately 50% of the macaque brain cortex and 20%–30% of the human brain cortex used for vision (Orban et al., 2004). The visual system is an ideal target for functional comparative research because the visual functional area of macaque brain is more developed than other regions. Vision is often used to study cognitive processes such as discrimination, attention, working memory and decision-making. Spyropoulos found that the parietal lobe in macaque can modulate the frequency encoding of visual input in V4, thereby regulating visual attention (Spyropoulos et al., 2018). Researchers also reported the role of visual cortex in retaining visual working memory information and working memory consolidation (Makovski and Lavidor, 2014). In addition, research in humans indicated that category-selective visual cortex is involved in the generalization process that facilitates reward transfer and memory-guided decision making (Liashenko et al., 2020). However, the functional connection patterns and anatomical connection patterns of the macaque visual cortex have not been investigated. Additionally, it is unclear whether humans and macaques have similar connectivity patterns. Therefore, it is necessary to conduct cross-species homology analysis of the visual system based on connectivity.

Previous studies have mentioned the brain regions comprising the visual system in the human brain. Volkmar found that the fusiform gyrus is involved in advanced visual processing of complex stimuli such as faces, especially object recognition and category recognition (Volkmar, 2021). Candidi found that the posterior superior temporal sulcus is involved in the conscious visual perception of emotional faces or bodies and is a key node in the visual system (Candidi et al., 2015). Cavanna demonstrated that the precuneus has functions such as visual spatial imagination, episodic memory retrieval and self-processing consciousness (Cavanna and Trimble, 2006), while Bruner supported this conclusion and found that the precuneus plays a crucial role in visual spatial integration (Bruner et al., 2017). Grill-Spector showed that the lateral occipital complex plays an important role in human recognition (Grill-Spector et al., 2001), while Ragni also found that the medial and lateral occipital cortices belong to the occipital lobe cortex and are closely related to vision (Ragni et al., 2020).

Previous studies have also involved the brain regions comprising the visual system in the macaque brain. Lu found that the V1 and V2 areas of the macaque visual cortex have a unique cytochrome oxidase staining pattern (Lu and Roe, 2008). Felleman found that V3 may involve certain aspects of shape analysis (Felleman and Van Essen, 1987), especially at low contrast levels. In another study (Felleman et al., 1997), the authors also showed that the convergence and divergence of the V3 pathway emphasize the distributed nature of hierarchical processing in the visual system. Nakamura found that V3A is a hyper-stripe visual area that provides the main input to the parietal cortex (Nakamura and Colby, 2000). The V4 area is a visual organization area in the macaque pre-lunate cortex and is a major source of visual input to the inferior temporal cortex, which is crucial for object recognition (Desimone and Schein, 1987). Additionally, the V4 area almost completely represents the binocular visual field, with the lower visual field representing the dorsal (V4d) and the upper visual field representing the ventral (V4v) (Pinon et al., 1998). In functional studies of monkeys, it was found that there are two visual areas in the caudal part of the superior parietal lobule (SPL): V6 and V6A (Gamberini et al., 2009). V6A is a visual motion area that contains neurons that respond to visual, somatic, oculomotor, and reaching stimuli, as well as cells that are modulated by animal attention levels. V6Ad is part of the prefrontal loop that controls prehension. Passarelli showed that V6Av is primarily a visual area that supports the dorsal cortical network and performs rapid form and motion analysis required for visually guided actions.

Psychophysical studies have shown that many aspects of visual perception in humans and macaques are very similar (Shushruth et al., 2013). Currently, cross-species research on the visual system of humans and macaques mainly focuses on cytoarchitecture, neurophysiology and comparative neuroimaging. Among them, cross-species studies of the visual system based on comparative neuroimaging are mainly divided into three categories: single-modality imaging research on a single brain area (Pitzalis et al., 2013), multimodality imaging research on a single brain area (Wang et al., 2019), and single-modality imaging research on partial visual cortex (Tosoni et al., 2015). Therefore, the lack of research on quantifying homology of the entire visual system based on multimodal imaging has resulted in a lack of clear understanding of the homology of the visual systems of humans and macaques. However, the combination of diffusion tensor imaging (DTI) and resting-state functional magnetic resonance imaging (RS-fMRI) helps bridge the gap of neurophysiology and imaging research between humans and macaques. Comparative functional MRI research on humans and macaques helps directly compare brain structures, from sensory systems to higher cognitive functions in association areas. Evidence from comparative functional MRI research, as well as evidence from cytoarchitectonic (Rapan et al., 2022) and neural connectivity, helps infer the homogeneity of given brain regions in humans and macaques. Using DTI to calculate the structure connection fingerprints is an important method to directly compare neural connections between humans and macaques (Mars et al., 2016) that can supplement comparative functional imaging research. The visual systems of humans and macaques have many similarities in anatomical and functional organization. Understanding the homologies and differences between the visual systems of the two species can provide important insights into the neural basis of visual perception and cognition.

In this research, our team constructed structural connectivity fingerprints and functional connectivity fingerprints for humans and macaques based on multimodal imaging of DTI and RS-fMRI to compare the structural and functional connectivity of the visual systems of the two species. The homology was then quantitatively analyzed using cosine similarity. The research revealed the homologous organization of the visual systems between the two species and established a visual system homology information map. This research provides new insights into the neural basis of visual perception and cognition and may provide a reference for the treatment of visual disorders, as well as laying the foundation for subsequent cross-species research.

## 2. Materials and methods

### 2.1. Dataset

#### 2.1.1. Human data

The human brain imaging data were obtained from the WU-Minn public dataset (Van Essen et al., 2013) of the Human Connectome Project (HCP) released by the HCP Data Center in 2016. After data cleaning to remove extreme values or missing data, 20 healthy subjects were selected, including 10 males and 10 females, aged 22-35 years. The selected brain imaging data included DTI, structural magnetic resonance imaging (sMRI), and RS-fMRI data. The diffusion MRI data was composed of three shells with 270 diffusion directions evenly distributed among them (b-values: 1,000, 2,000, and 3,000 s/mm^2^) and six b = 0 s/mm^2^ acquisitions inside each shell. The DTI data included multigradient 1.25 mm isotropic data and the sMRI data included 0.7 mm high resolution isotropic T1-weighted (T1w) and T2-weighted (T2w) imaging data. The data were acquired using a standard 32-channel Siemens head coil and a special gradient body transmission coil on a Siemens Skyra scanner in Germany. The diffusion MRI data acquisition parameters were TR = 5,520 ms, TE = 89.5 ms, and voxel resolution = 1.25 × 1.25 × 1.25 mm. The T1w imaging acquisition parameters were TR/TE = 2,400/2.14 ms, flip angle = 7°, and voxel resolution = 0.7 × 0.7 × 0.7 mm. The T2w imaging voxel resolution was 0.3 × 0.3 × 0.3 mm, TE = 307 ms, and TR = 3,000 ms. The entire experiment was carried out in accordance with the Helsinki Declaration.

#### 2.1.2. Macaque data

The macaque brain imaging data were obtained from the public dataset of the PRIMatE Data Exchange (PRIME-DE) project (Milham et al., 2018). 20 subjects were selected from the University of California, Davis (UC-Davis), aged 18.5–22.5 years and weighing 7.28–14.95 kg. The selected brain imaging data included DTI, sMRI and RS-fMRI data. The DTI data acquisition parameters were TE = 115 ms, TR = 6,400 ms, diffusion-weighted b values of 1,600 s/mm^2^ and 800 s/mm^2^. The T1w imaging acquisition parameters were TE = 3.65 ms, TR = 2,500 ms, TI = 1100 ms, flip angle = 7°, and voxel resolution = 0.3 × 0.3 × 0.3 mm. The RS-fMRI data acquisition parameters were voxel resolution = 1.4 × 1.4 × 1.4 mm, TE = 24 ms, and TR = 1,600 ms. The macaques were kept in compliance with the UC-Davis IACUC ethical certification. The data acquisition experiments were approved by the local ethics committee and complied with the EU Directive on the protection of animals used for scientific purposes (2010/63/EU).

### 2.2. Diffusion MRI data preprocessing

The diffusion MRI data were preprocessed using FSL software (Smith et al., 2004), including six steps: (1) Head motion correction: use the EDDY tool to correct head motion in diffusion MRI (Andersson and Sotiropoulos, 2016); (2) Gradient direction correction: use the FDT tool to correct eddy currents in diffusion MRI data; (3) EPI distortions correction: use the TOPUP tool provided by FSL to correct the EPI image distortions (Andersson et al., 2003); (4) Brain tissue extraction: our researcher used the ResTLU-net tool developed by our team to extract brain tissue files from T1 and diffusion images and remove nonbrain tissue (Wang et al., 2022a); (5) Two-step registration: use the two-step registration method developed by our team to register individual image space and atlas template space; perform alignment, including individual image space alignment and atlas template space alignment (Wang et al., 2022b); (6) Adopt the BEDPOSTX two-tensor model provided by the FMRIB toolbox to estimate the dispersion parameters (Jbabdi et al., 2012).

### 2.3. Resting-state fMRI data preprocessing

The RS-fMRI data were processed using DPABI software (Yan et al., 2016), including six steps: (1)Remove the first 10 time points; (2) Slice timing: select the middle layer as the reference and align the other layers to it; (3) Head motion correction: exclude subjects with head motion parameters greater than 0.2; (4) Spatial normalization: the image space was normalized to the Montreal Neuropathy Institute(MNI) space, with the human brain resampled at 3 × 3 × 3 mm voxels and the monkey brain resampled at 1.375 × 1.375 × 1.375 mm voxels; (5) Filtering: set the filtering parameters to 0.01–0.1 Hz; (6) Smoothing: use a Gaussian kernel with a full-width-at-half-maximum (FWHM) of 6 mm to smooth the space after spatial normalization.

### 2.4. Regions of interest selection for the visual system of humans and macaques

#### 2.4.1. Visual brain regions in the human brain

Based on previous studies on brain regions of the human visual system, the research team proposed that the human visual system mainly includes brain regions such as the fusiform gyrus, posterior temporal sulcus, precuneus, medial and lateral occipital cortices, etc., which correspond to the region names in the Brainnetome Atlas (Fan et al., 2016) of the Chinese Academy of Sciences(CAS) and other detailed information, as shown in Table 1.

**Table 1:** Numbering of brain regions of the visual system in the human brain in the atlas.

**Name of the area in the map**	**Abbreviations**	**MNI(L)**	**MNI(R)**	**Volume(mm^3^)**
Rostroventral area 20	A20rv	[-33,-16,-32]	[33,-15,-34]	17,024
Medioventral area 37	A37mv	[-31,-64,-14]	[31,-62,-14]	13,600
Lateroventral area 37	A37lv	[-42,-51,-17]	[43,-49,-19]	14,736
Rostroposterior superior temporal sulcus	rpSTS	[-54,-40,4]	[53,-37,3]	5,360
Caudoposterior superior temporal sulcus	cpSTS	[-52,-50,11]	[57,-40,12]	5,144
Medial area 7(PEp)	A7m	[-5,-63, 51]	[6,-65, 51]	7,736
Medial area 5(PEm)	A5m	[-8,-47, 57]	[7,-47, 58]	9,976
Dorsomedial parietooccipital sulcus	dmPOS	[-12,-67, 25]	[16,-64, 25]	15,400
Area 31 (Lc1)	A31	[-6,-55, 34]	[6,-54, 35]	13,632
Caudal lingual gyrus	cLinG	[-11,-82,-11]	[10,-85,-9]	8,760
Rostral cuneus gyrus	rCunG	[-5,-81, 10]	[7,-76, 11]	12,976
Caudal cuneus gyrus	cCunG	[-6,-94, 1]	[8,-90, 12]	9,896
Rostral lingual gyrus	rLinG	[-17,-60,-6]	[18,-60,-7]	12,968
Ventromedial parietooccipital sulcus	vmPOS	[-13,-68, 12]	[15,-63, 12]	16,144
Medial superior occipital gyrus	msOccG	[-31,-89, 11]	[34,-86, 11]	13,560
Lateral superior occipital gyrus	lsOccG	[-46,-74, 3]	[48,-70,-1]	12,464
Middle occipital gyrus	mOccG	[-18,-99, 2]	[22,-97, 4]	15,888
Area V5/MT+	MT+	[-30,-88,-12]	[32,-85,-12]	15,752
Occipital polar cortex	OPC	[-11,-88, 31]	[16,-85, 34]	10,160
Inferior occipital gyrus	iOccG	[-22,-77, 36]	[29,-75, 36]	10,792

#### 2.4.2. Visual brain regions in the macaque brain

Based on previous studies on brain regions of the macaque visual system, the research team proposed that the macaque visual system mainly includes brain regions such as V1, V2, V3A, V3d, V3v, V4, V4v, V6, V6Av, and V6Ad. These brain regions correspond to the region names in the D99 macaque brain atlas (Reveley et al., 2017) and other detailed information, as shown in Table 2.

**Table 2:** Numbering of brain regions of the visual system in the monkey brain in the atlas.

**Brain areas**	**Name of the area in the map**	**MNI coordinates**	**Volume(mm^3^)**
V1	Visual area 1 (primary visual cortex)	[1.19,-37.18,8.20]	5,032
V2	Visual area 2	[8.26,-31.61,6.07]	4,718
V3A	Visual area V3A	[0.99,-29.03,13.70]	317
V3d	Visual area 3, dorsal part	[1.84,-30.84,13.39]	338
V3v	Visual area 3, ventral part	[5.16,-26.82,-1.90]	577
V4	Visual area 4 (dorsal part)	[-9.21,-24.95,14.02]	1,296
V4v	Visual area 4, ventral part	[11.80,-23.56,-3.61]	602
V6	Visual area 6	[2.24,-30.91,11.03]	94
V6Ad	Visual area 6A, dorsal division	[11.28,43,19.57]	79
V6Av	Visual area 6A, ventral division	[-8.06,-30.57,15.42]	95

Referring to the homologous point coordinates provided by Neubert et al. (2015) and combining with the selected brain atlas registered to the space of the coordinates, the research obtain the brain area based on the coordinate points and extract the brain area as the target region. Our team identified 18 homologous brain regions as target regions. The target regions included 9/46d, region 44v, supplementary motor cortex (SMA), region 8 medial (8m), primary motor cortex (M1), primary somatosensory cortex (S1), parietal insula, posterior intraparietal sulcus, posterior inferior parietal lobule, region 23ab, posterior pressure cortex, perinasal cortex, ventral striatum, hippocampus, parahippocampal gyrus, dorsomedial prefrontal lobe, dorsolateral prefrontal lobe, and insula (Wang et al., 2022b), as shown in Table 3. The relevant regions of interest (ROIs) were manually extracted using FSL software on the two atlases.

**Table 3:** The coordinates of the homologs.

**Brain areas**	**Abbreviations**	**Human MNI space**	**Macaque MNI space**
Region 9/46 dorsal	9/46d	[42,36,38]	[-12.75,11.5,9.25]
Region 44v	44v	[52,20,8]	[-17,7.5,1.5]
Supplementary motor cortex	SMA	[4,-4,60]	[-2,-0.5,18]
Region 8 medial	8m	[-4,36,56]	[-1.75,16.75,13.25]
Primary motor cortex	M1	[-42,-18,48]	[-13.25,-6.5,16]
Primary somatosensory cortex	S1	[-44,-30,58]	[-12.25,-9.5,18.5]
Parietal insula	7op	[-52,-30,22]	[-18,-14.75,12.5]
Posterior intraparietal sulcus	pIPS	[-34,-48,42]	[-9.75,-16.75,17.75]
Posterior inferior parietal lobule	pIPL	[-20,-76,44]	[-8,-24,16.25]
Region 23ab	23b	[-2,-20,36]	[0.25,-8.5,12.75]
Posterior pressure cortex	rsplC	[-8,-48,10]	[-2.5,-17,3.75]
Perinasal cortex	perirhinal	[-46,0,-38]	[-16,-3.75,-13.25]
Ventral striatum	ventrStr	[-14,10,-10]	[-3.5,6.5,-3]
Hippocampus	hippoc	[-26,-20,-16]	[11,-4,-11.25]
Parahippocampal gyrus	35/36r	[-27,-7,-34]	[-12.5,-6,-16]
Dorsomedial prefrontal lobe	9m	[-5,36,38]	[-1,23,8.5]
Dorsolateral prefrontal lobe	8dl	[-18,24,53]	[-3.5,17.5,15]
Granular insula	Ig	[-38,-8,8]	[-19,-7,3.5]

### 2.5. Establishing the visual system homology information map

#### 2.5.1. Anatomical connectivity analysis

Probabilistic tractography was performed using PROBTRACKX, provided with the FMRIB software package, for each voxel in each ROI from humans and macaques emitting 5000 streamlines to the target region. To compensate for the lower gradient magnetic field applied to the macaque data, 50,000 streamlines were emitted from each voxel in the macaque ROIs to the target region for probabilistic fiber tracing. The tracking values were averaged and then normalized using maximum-minimum normalization to obtain the structural connectivity matrices of the human and macaque brains. The similarity between the constructed structural connectivity matrices of the human and macaque brains was quantified using the cosine similarity to obtain the structural similarity matrix (Wang et al., 2022b), and the permutation test was used to verify the reliability of the experimental conclusions. By calculating the cosine similarity between pairs of brain regions, we can assess the degree of similarity in their structural connectivity patterns. A higher cosine similarity score suggests that the connectivity profiles of two regions are more similar, while a lower score indicates greater dissimilarity. By performing 10,000 permutations of the sample order, the test statistic was recalculated, and an empirical distribution was constructed to assess its credibility. The empirical distribution was then subjected to 1,000 repetitions of the test to obtain a *p*-value. A significance level of *p* < 0.05 was considered indicative of statistically significant differences.

#### 2.5.2. Functional connectivity analysis

The BOLD time series of the human and macaque brain ROIs were extracted from the resting-state data after preprocessing using the DPABI toolkit. The Pearson correlation coefficients between each pair of time series were calculated. The correlation coefficients were defined as the connection strength values after Fisher Z-transformation, and then the maximum-minimum normalization was performed to obtain the functional connectivity matrices of the human and macaque brains. The similarity between the functional connectivity matrices of the human and macaque brains was quantified using the cosine similarity to obtain the functional similarity matrix (Wang et al., 2022b), and the reliability of the experimental findings was verified using the permutation test. The settings for the permutation test were the same as those used in the anatomical connectivity analysis.

#### 2.5.3. Establishing homology information map

**Figure 1:**
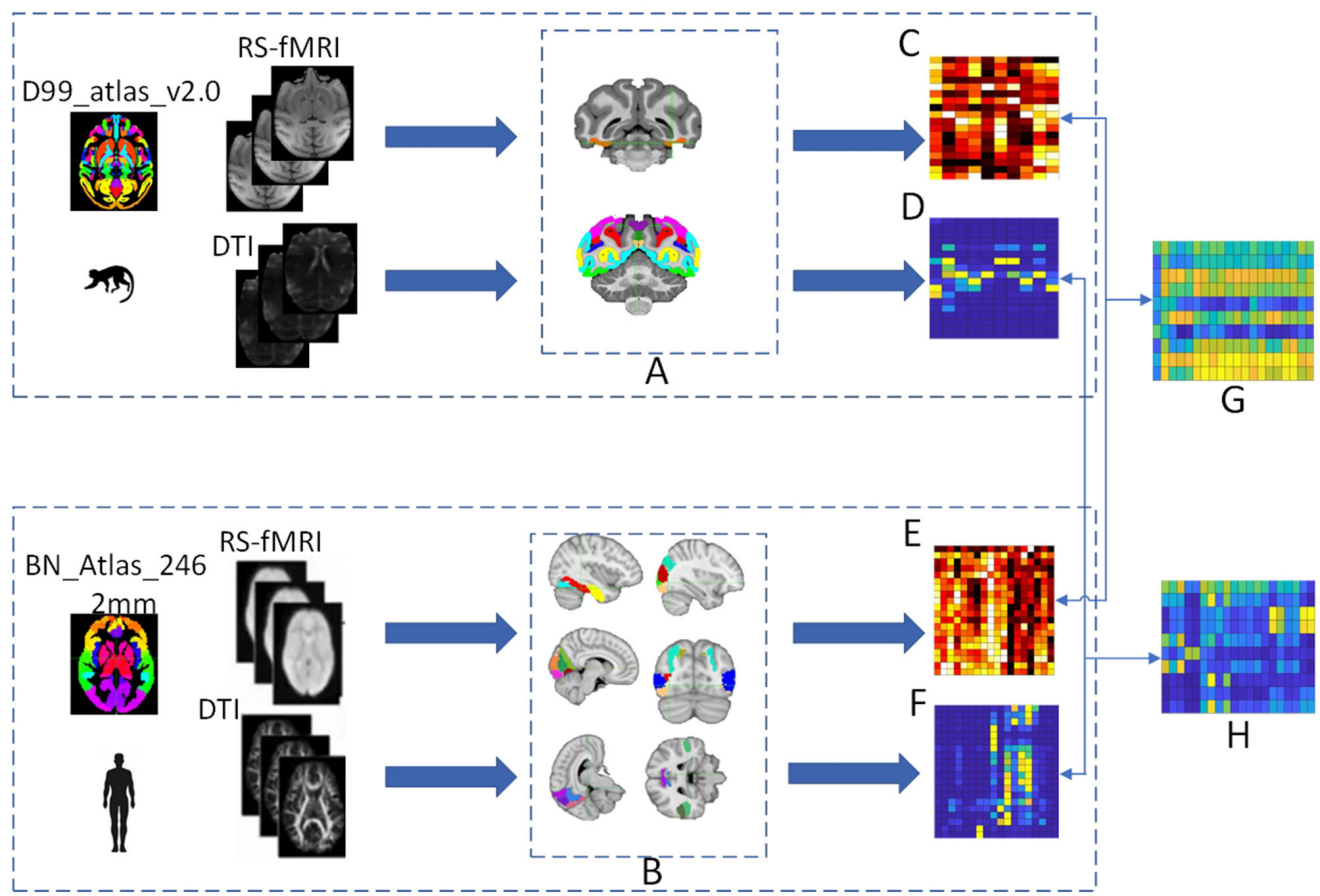
The process of establishing the homologous information map. **(A)** Represents the position of the monkey visual system ROIs in the D99 template, including V1, V2, V3A, V3d, V3v, V4, V4v, V6, V6Av, and V6Ad. **(B)** Represents the position of the human brain regions of interest in the MNI template, including the fusiform gyrus, superior temporal sulcus, prefrontal cortex, and occipital cortex. Functional connectivity matrices are obtained for both human and monkey brains after preprocessing and time series extraction, with **(C)** representing the monkey functional connectivity matrix and **(E)** representing the human functional connectivity matrix. Structural connectivity matrices are obtained for both human and monkey brains after preprocessing and probabilistic fiber tractography, with **(D)** representing the monkey structural connectivity matrix and **(F)** representing the human structural connectivity matrix. The cosine similarity is used to quantitatively analyze the human-monkey structural and functional connectivity matrices to obtain similarity matrices, with **(G)** representing the human-monkey functional similarity matrix and **(H)** representing the human-monkey structural similarity matrix.

Based on the visual system brain areas of humans and macaques identified by the research team, probabilistic fiber tractography was used to calculate the structural connectivity between the ROIs and predefined homologous target regions, and seed-based correlation analysis was used to calculate the functional connectivity between the ROIs and predefined homologous target regions. Constructing connectivity fingerprints by taking the region of interest as the center, the homologous target regions as vertices, and the connectivity strength values as the radius. Then structural and functional connectivity fingerprint maps are generated to reveal their characteristics. The cosine similarity is used to quantitatively analyze the homology of the structural and functional connectivity matrices. Brain regions with similarity results greater than 0.6 for both types of connectivity matrices are selected as homologous brain regions, and the visual system homologous information map is established. The process for constructing the homologous information map is shown in Figure 1.

### 2.6. Reliability of the tests

To ensure reliability, we employed standardized protocols and procedures throughout the data acquisition and analysis processes. These protocols have been widely adopted and validated in the field of neuroimaging. Specifically, we followed established guidelines for data preprocessing, quality control, and statistical analysis. By adhering to these rigorous standards, we aimed to minimize potential sources of variability and enhance the reliability of our measurements.

## 3. Results

### 3.1. Cross-species comparisons of structural connectivity patterns

**Figure 2:**
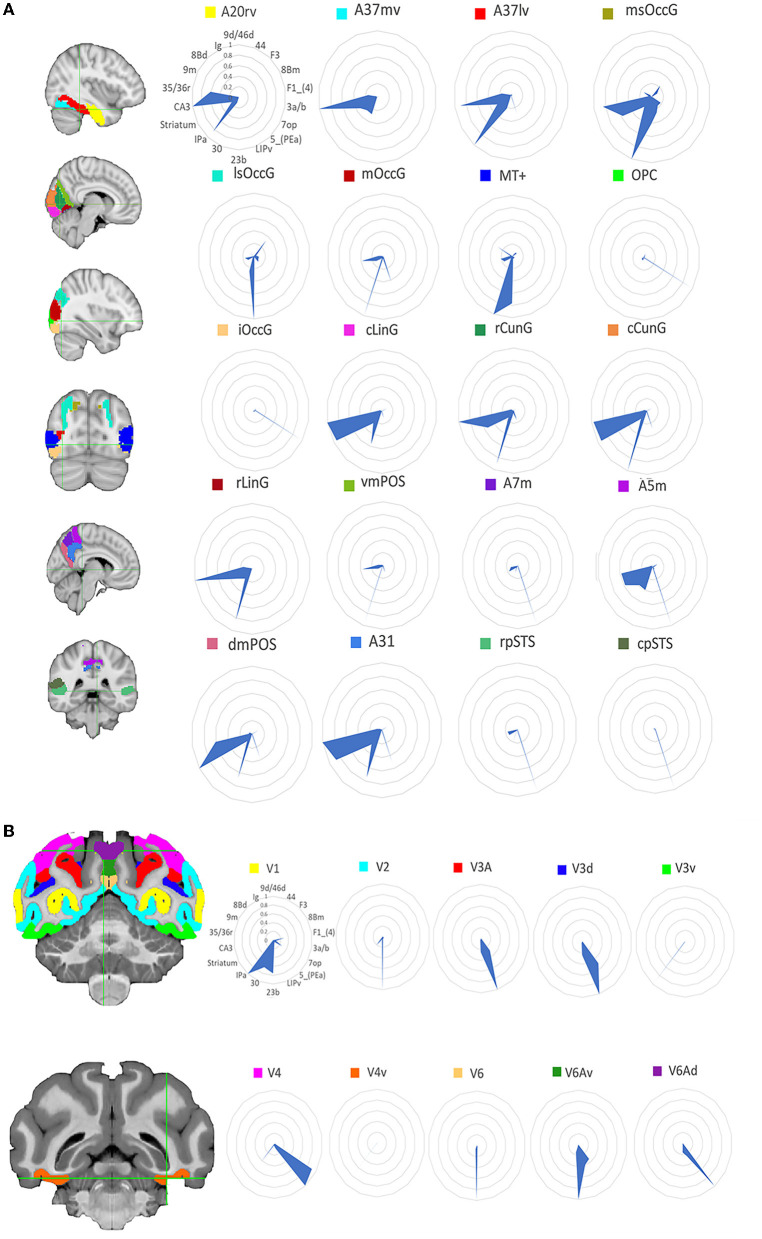
Structural connectivity fingerprint map. **(A)** Is the human visual system, which contains 20 brain regions, mainly located in the temporal lobe, occipital lobe, and precuneus. **(B)** Shows the monkey visual system, which includes 10 brain regions, mainly located in the occipital lobe. Different brain regions are shown in different colors, and the structural connectivity fingerprints of the ROIs are shown on the right. The corresponding abbreviations for the target regions in the monkey brain D99 atlas are: 9d/46d, 9/46d; 44, area 44v; F3, auxiliary motor cortex; 8Bm, region 8 medial; F1_(4), major motor cortex; 3a/b, major somatosensory cortex; 7op, parietal insula; 5_(PEa), posterior parietal sulci; LIPv, posterior parietal lobule; 23b, region 23ab; 30, posterior cortex; IPa, perineasal cortex; Striatum, ventral striatum; CA3, hippocampus; 35/36r, parahippocampal gyrus; 9m, dorsolateral prefrontal cortex; 8Bd, dorsolateral prefrontal cortex; Ig, granular insula.

**Figure 3:**
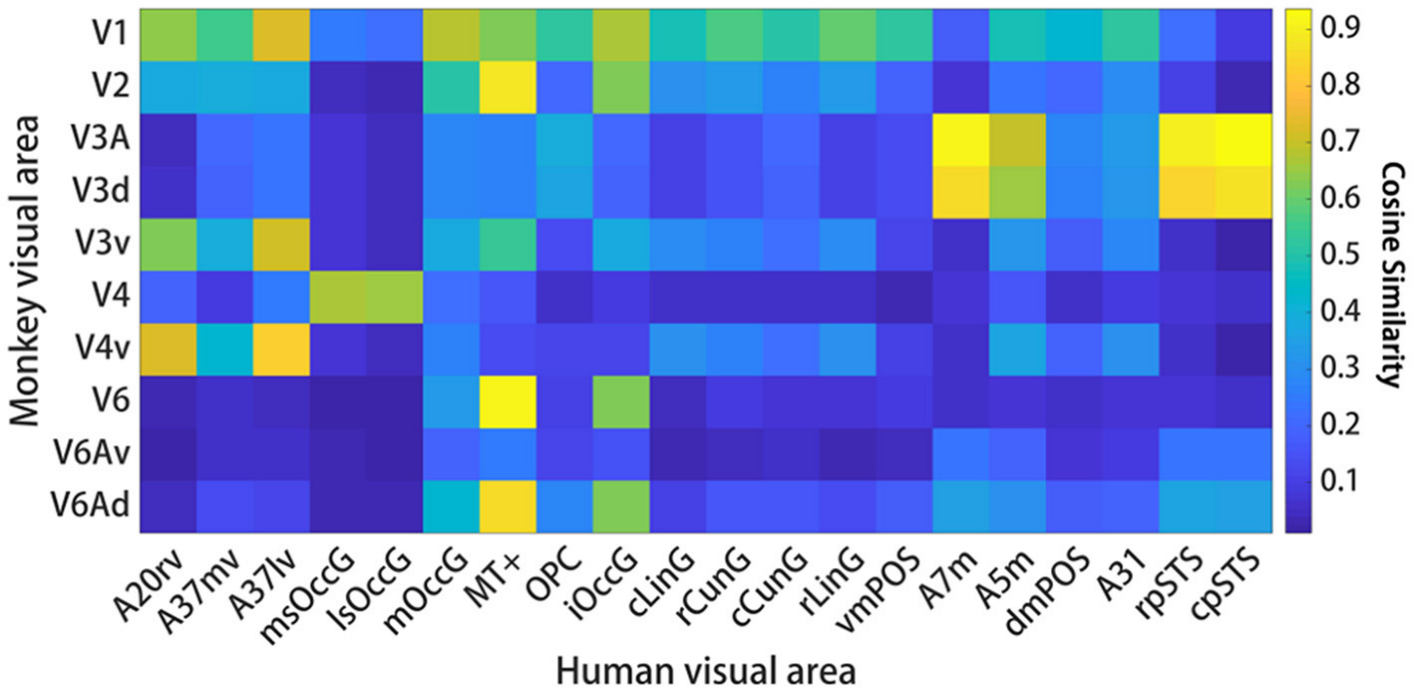
Interspecies structural connectivity similarity. For each species, cosine similarity values are plotted in matrix form. A high cosine similarity value suggests that the connectivity values are more comparable than a lower cosine similarity value. The results show that there are 25 pairs of human and macaque brain regions with similarity greater than 0.6. Additionally, an intra-species structural similarity analysis was performed between the human and monkey brains (see Supplementary Figures 5, 6), and the results demonstrated that both species exhibited intra-species consistency.

The human tracking results is in the Supplementary Figure 1 and the macaque tracking results is in the Supplementary Figure 2. To compare the structural connectivity (SC) patterns between humans and macaques, researchers computed the SC fingerprints of 20 visual ROIs in humans and 10 visual ROIs in macaques. Then we computed cosine similarities for pairs of fingerprints. Figure 2 shows the location of these regions and the corresponding normalized SC fingerprints for humans and macaques. In order to compare the structural connections of the human and macaque visual systems, the cosine similarity was used to calculate the similarity of the two species' structural connections. The results (Figure 3) showed that there were 25 pairs of human and macaque brain regions with similarity greater than 0.6. The research results indicate that the human and macaque visual systems have regional homology in structural connections, and there was no significant difference between the two groups compared by permutation test.

### 3.2. Cross-species comparisons of functional connectivity patterns

**Figure 4:**
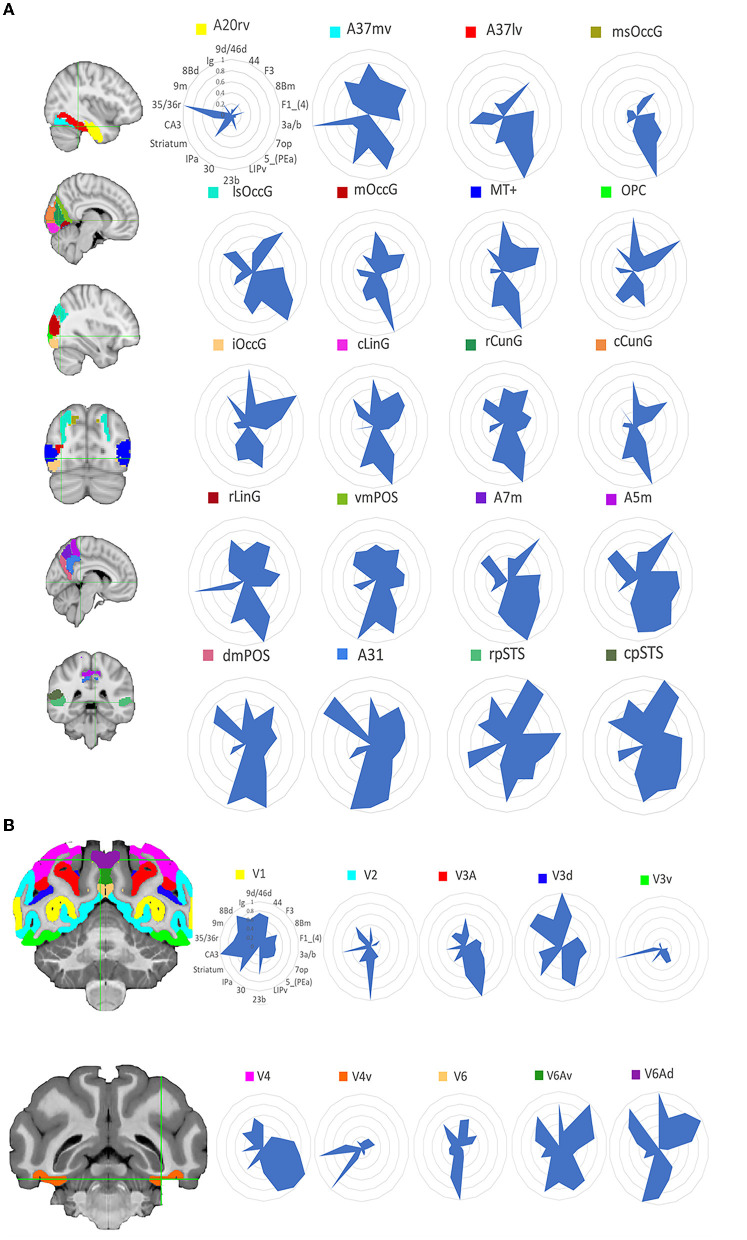
Functional connectivity fingerprint map. **(A)** Shows the human visual system and **(B)** shows the monkey visual system. Different brain regions are shown in different colors, and the functional connection fingerprint of the region of interest is shown on the right, with the corresponding abbreviation in the D99 macaque brain atlas, as shown in Figure 2.

**Figure 5:**
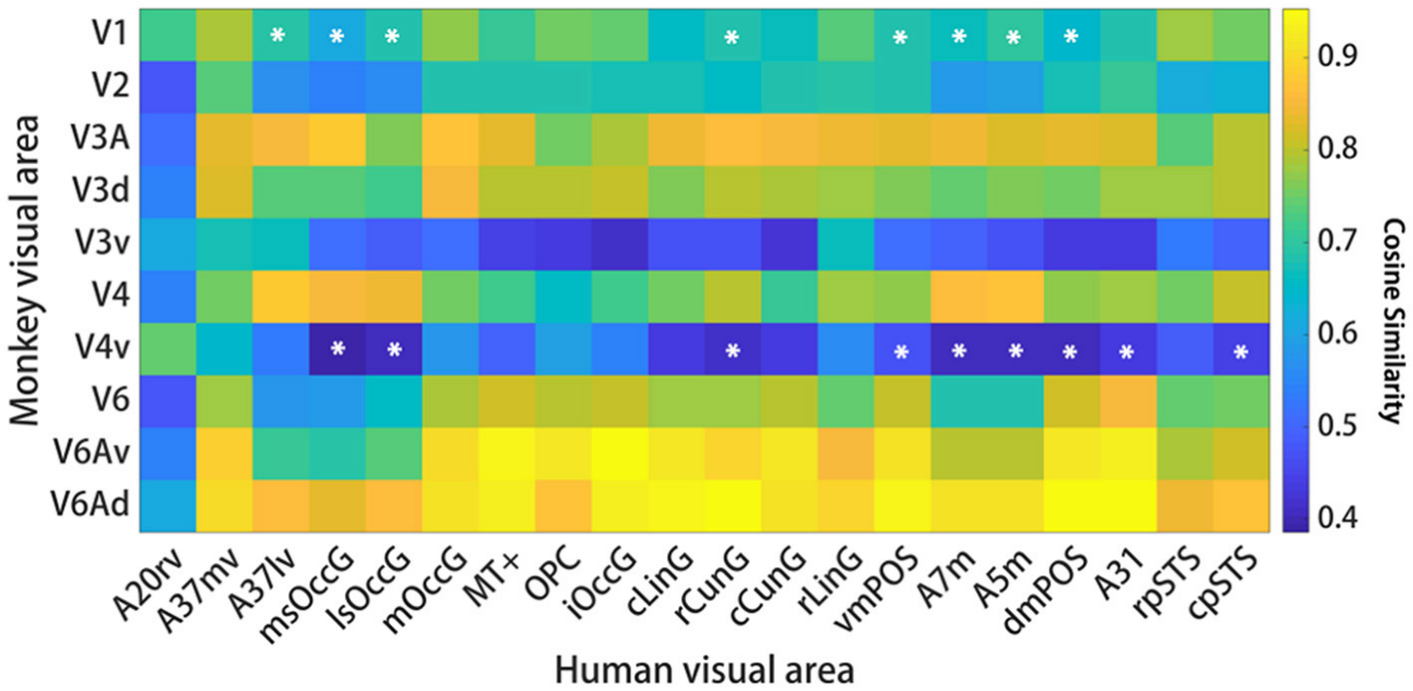
Interspecific functional connectivity similarity. For each species, cosine similarity values are plotted in matrix form. A high cosine similarity value suggests that the connectivity values are more comparable than a lower cosine similarity value. Significant differences are marked by a white asterisk within the similarity matrix. The results suggest that the interspecific functional similarity is high and that the species are functionally homologous.

The human functional connectivity data is in the Supplementary Figure 3 and the macaque functional connectivity data is in the Supplementary Figure 4. For comparisons of visual functional connectivity (FC) patterns between the two species, researchers computed the FC fingerprints of 20 visual ROIs in humans and 10 visual ROIs in macaques. Then we computed cosine similarities for pairs of fingerprints. Figure 4 shows the location of these regions and the corresponding normalized FC fingerprints for humans and macaques. To compare the FC fingerprints between the two species, we computed cosine similarities for pairs of fingerprints. As shown in Figure 5, the comparison of FC fingerprints between human and macaque fingerprints revealed lots of the region pairs (153/200) with cosine similarity metrics higher than 0.6, indicating similar FC profiles in the visual system between humans and macaques. Several comparisons were still significantly different between the two species (17/200; *p* < 0.05).This finding shows that the macaque visual cortex FC fingerprints resemble those of the human visual cortex.

### 3.3. Homology information map of the visual system

**Figure 6:**
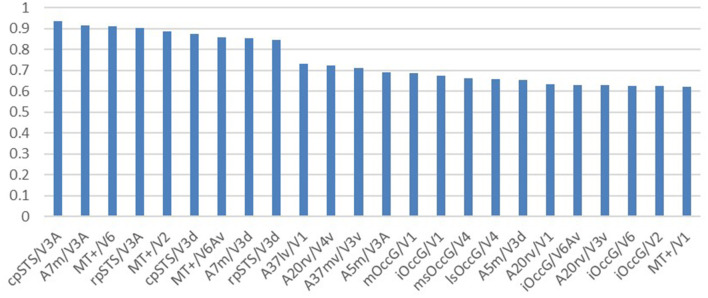
Structural similarity ranking of homologous brain regions. The vertical coordinate is the structural cosine similarity, and the horizontal coordinate is the name of the brain region. The left side of the “/” is the human visual brain region and the right side of the “/” is the macaque visual brain region. The highest similarity is found in the cpSTS brain region in humans and the V3A brain region in macaques.

**Figure 7:**
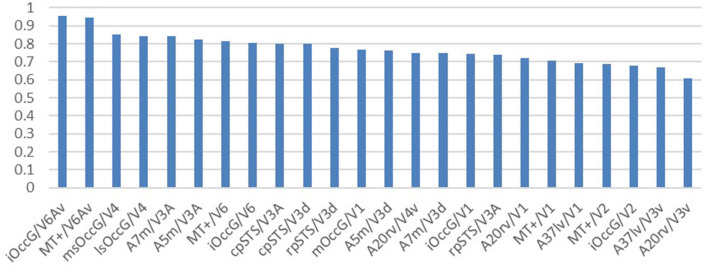
Functional similarity ranking of homologous brain regions. The vertical coordinate is the functional cosine similarity and the horizontal coordinate is the name of the brain region, with the human visual brain region to the left of the “/” and the macaque visual brain region to the right of the “/”. The highest similarity is reported for the iOccG brain region in humans and the V6Av brain region in macaques.

**Figure 8:**
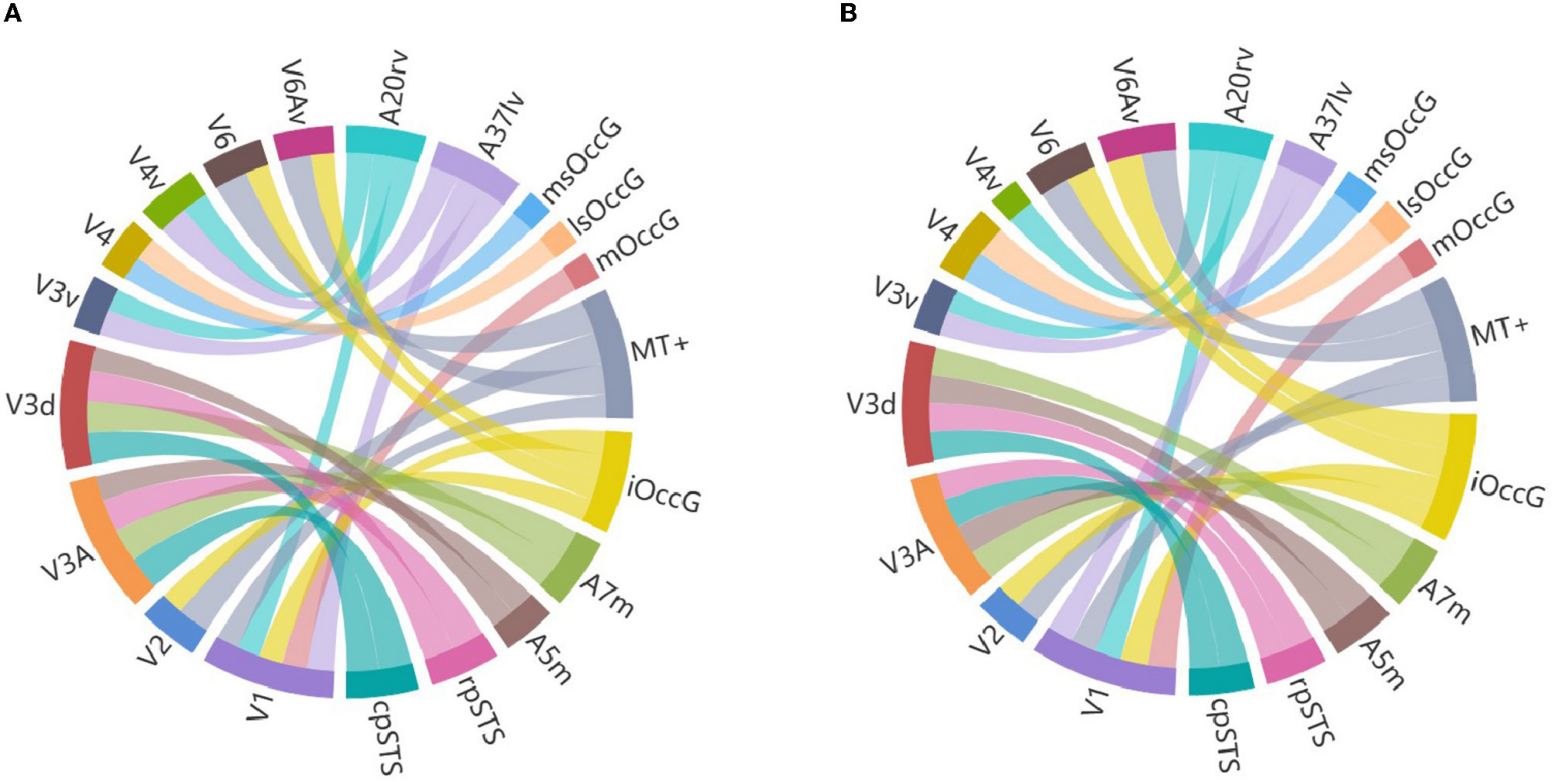
Visual system homology information map. **(A)** Is a structural homology information map, and **(B)** is a functional homology information map, where V1, V2, V3A, V3d, V3v, V4, V4v, V6 and V6Av are macaque visual brain regions, A20rv, A37lv, msOccG, lsOccG, mOccG, MT+, iOccG, A7m, A5m, rpSTS and cpSTS are human visual brain regions, with different brain regions represented by different colors and the thickness of the lines representing the degree of similarity between brain regions.

Researchers selected brain regions with similarity scores >0.6 from both the structural and functional similarity matrices to identify homologous brain regions. Table 4 shows the 24 selected homologous brain regions. As shown in Figure 6, the structural connectivity pattern of the macaque V3A area is most similar to the human cpSTS area, and both V3A and cpSTS exhibit strong connectivity with the LIPv area. V3A is located in the occipital visual cortex in macaques, while cpSTS is adjacent to the occipital lobe in the human brain, and LIPv is located in the posterior inferior parietal lobule. Studies have shown that the topological connections between human and macaque frontal areas are similar (Rushworth et al., 2006), and some anatomical studies have emphasized the similarities between the human and macaque inferior parietal lobules (Eidelberg and Galaburda, 1984), which may explain the stable connection between V3A and cpSTS. As shown in Figure 7, the functional connectivity pattern of the macaque V4 area is most similar to that of the human lsOccG area. V4 is located in the temporal lobe gyrus in macaques, while lsOccG is located on the lateral surface of the occipital visual cortex in humans, and the occipito-temporal connections in humans and monkeys are highly similar (Catani et al., 2003). Moreover, the last part of multiple retinotopic maps in the ventral surface of the human occipital lobe is considered a potential homology of macaque V4 (Winawer and Witthoft, 2015). Therefore, the high similarity between V4 and lsOccG is reliable. Based on these findings, the researchers drew a homology information map of the human and macaque visual systems (Figure 8) to summarize the homologous and differential structural and functional connections between brain regions.

**Table 4:** Homologous brain region pairs in the human and monkey visual systems.

**Humanity**	**Macaque**	**Structural similarity**	**Functional similarity**
A20rv	V1	0.633	0.719
A37lv	V1	0.733	0.693
mOccG	V1	0.685	0.767
MT+	V1	0.623	0.707
iOccG	V1	0.673	0.745
MT+	V2	0.886	0.687
iOccG	V2	0.625	0.678
A7m	V3A	0.915	0.841
A5m	V3A	0.692	0.821
rpSTS	V3A	0.903	0.740
cpSTS	V3A	0.936	0.801
A7m	V3d	0.854	0.746
A5m	V3d	0.653	0.761
rpSTS	V3d	0.844	0.776
cpSTS	V3d	0.874	0.801
A20rv	V3v	0.627	0.608
A37lv	V3v	0.709	0.669
msOccG	V4	0.662	0.850
lsOccG	V4	0.657	0.844
A20rv	V4v	0.722	0.747
MT+	V6	0.912	0.813
iOccG	V6	0.627	0.806
MT+	V6Av	0.859	0.943
iOccG	V6Av	0.628	0.952

## 4. Discussion

Researchers identified the visual brain regions in humans and macaques based on previous studies. Using the Brainnetome Atlas of the CAS and the D99 Atlas of the macaque, our team extracted the visual brain regions and constructed structural and functional connectivity fingerprints. Then we quantitatively analyzed the fingerprint graphs using cosine similarity to obtain structural and functional similarity matrices. We selected brain regions with high similarity in both matrices as homologous regions, and found that the functional organization of the visual system between the two species is conserved and homologous. There are 24 homologous visual brain regions between humans and macaques, and homologous information maps were created for these regions. This research lays the foundation for future cross-species research.

V1 and V2 are located in the visual cortex of the occipital lobe of the macaque brain, while mOccG, MT+ and iOccG are located in the lateral occipital cortex of the occipital lobe of the human brain. The homologous information map shows that V1 and V2 are structurally and functionally homologous to MT+ and iOccG, and that V1 is structurally and functionally homologous to mOccG. This is consistent with the findings of Van Essen et al. who found many commonalities in functional organization between humans and various nonhuman primates, especially in the visual cortex of the occipital lobe (Van Essen et al., 2001). Research has found that the adjacent and overlapping areas of the macaque anterior STS/MTG have strong responses to facial and body recognition, similar to the areas of the human fusiform gyrus and posterior STS (Pinsk et al., 2009), indicating a high degree of similarity between the human and macaque fusiform gyrus. The homologous information map shows that the V4v area of the macaque fusiform gyrus is homologous to the A20rv area of the human fusiform gyrus, which is consistent with previous research results. Gallant found that the retinotopically organized region outside the human visual cortex is functionally homologous to the macaque V4v area (Gallant et al., 2000), enhancing the credibility of the homologous relationship between the human and macaque fusiform gyrus. Catani found that the temporal lobe connections identified by DTI in humans are very similar to the connections described in the macaque temporal lobe (Catani et al., 2003). This is also reflected in the homologous information map. The homologous information map shows that the V4 area of the macaque temporal lobe fusiform gyrus is homologous to the msOccG and lsOccG areas of the human occipital cortex. Rushworth found that the human parietal lobe has anatomical connections similar to the functionally related macaque parietal lobe (Rushworth et al., 2006). Rottschy found that the structure and functional organization of the V1, V2, V3A, V3d, V3v, and V4 brain regions in macaques are largely preserved in humans (Rottschy et al., 2007), which is consistent with the 24 homologous visual brain regions we identified, enhancing the credibility of the homologous information map. Based on these findings, the research team believes that the homologous information map of the human and macaque visual systems is credible.

These findings have important implications for our understanding of various aspects of brain function. Regarding neurodevelopment, the identification of homologous brain regions between species provides insights into the evolutionary conservation of neural circuits involved in visual processing (Rakic, 2009). Understanding the developmental trajectories and molecular mechanisms underlying the establishment of these homologous connections can shed light on the principles governing brain development and the emergence of visual functions in both humans and macaques. In terms of cortical organizational principles, our findings support the concept of functional homology between species, suggesting that certain brain regions involved in visual processing maintain similar functions across primate evolution (Sereno and Tootell, 2005). This highlights the importance of comparative studies in elucidating the principles underlying the organization and specialization of the visual system. Furthermore, the identification of homologous brain regions between humans and macaques has implications for understanding brain diseases and disorders. By studying the similarities and differences in connectivity patterns between species, we can gain insights into the neural mechanisms underlying visual impairments (Goodale and Melvyn, 2013) and potentially identify targets for therapeutic interventions.

## 5. Conclusion

This research explored the homology of human and macaque visual areas in terms of structure and function based on connectivity. The results show that the visual systems of both species are regionally homologous in structure and functionally homologous in organization. Our team identified 24 homologous visual brain regions between humans and macaques and created homologous information maps for these regions, providing theoretical support for future cross-species comparative frameworks and references for the development of new treatments for visual impairments. Although some progress has been made, the sample size of the human and macaque data used in this study is small after data processing and screening. Future research can expand the data, use higher computing power devices, and verify the results using different datasets to improve cross-species comparative research. Furthermore, after expanding the sample size, variables such as gender and geography can be controlled to investigate their effects on the evolution of the brains of both species and the resulting interspecies differences.

## Data availability statement

The original contributions presented in the study are included in the article/Supplementary material, further inquiries can be directed to the corresponding author.

## Ethics statement

The studies involving human participants were reviewed and approved by special member of the Ethics Committee of the Academic Committee of Taiyuan University of Technology. The patients/participants provided their written informed consent to participate in this study. The animal study was reviewed and approved by UC-Davis IACUC.

## Author contributions

HL and QW evaluated and guided the experimental design of this research. XL conceived and designed the experiments and wrote the manuscript. XWL and GW deal with the data of structure and function. YC collected the data. XQL analyzed the results. All authors contributed to the article and approved the submitted version.
