# Supplementary material for: Connectivity reveals homology between the visual systems of the human and macaque brains
Source: Front Neurosci. 2023 Jul 5;17:1207340. doi: 10.3389/fnins.2023.1207340 (PMC10354265; doi:10.3389/fnins.2023.1207340)
Supplement: Supplementary file 1 [file Data_Sheet_1.docx]

Supplementary Material

# Supplementary Figures


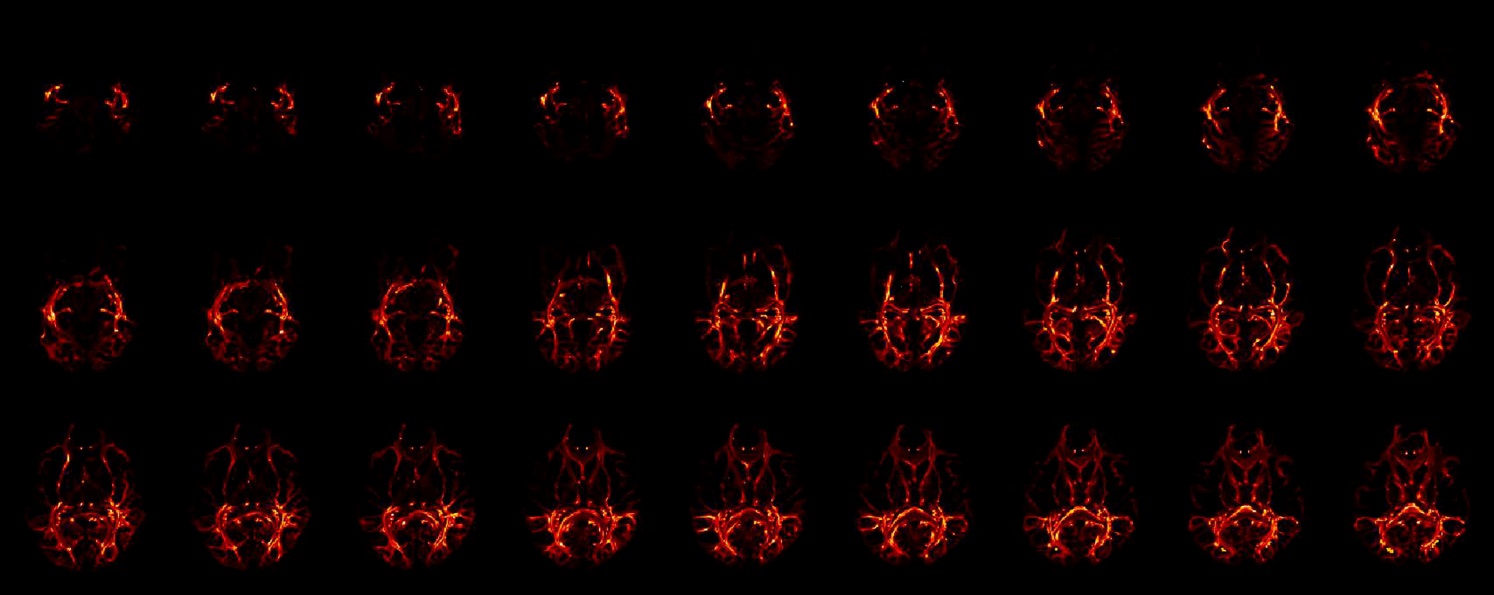


**Supplementary Figure 1.** Tracking results of the human subject. Probabilistic tractography was performed using PROBTRACKX on human diffusion MRI data. Each ROI from humans emitting 5,000 streamlines to the target region. The color-coded streamlines represent the estimated trajectories of the fiber bundles connecting different ROIs within the human brain. The resulting streamlines were visualized using FSLeyes.


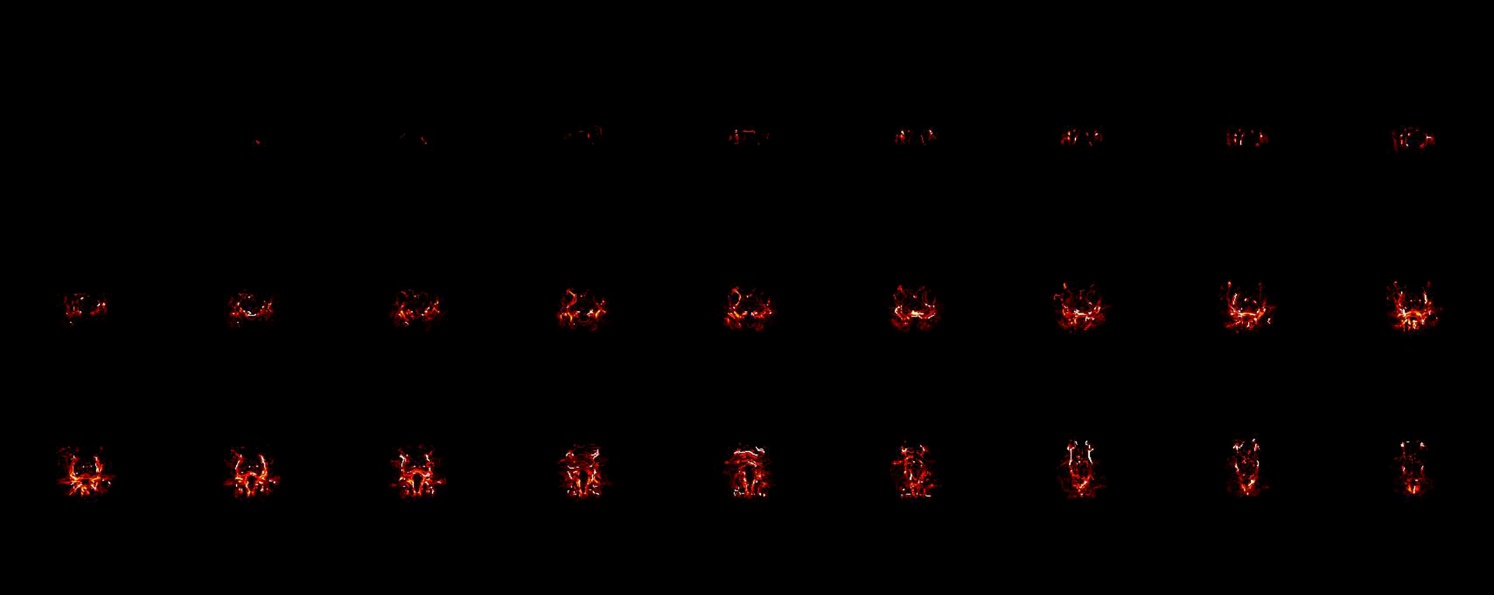


**Supplementary Figure 2.** Tracking results of the macaque subject. Probabilistic tractography was performed using PROBTRACKX on macaque diffusion MRI data. Each ROI from macaques emitting 50,000 streamlines to the target region. The color-coded streamlines represent the estimated trajectories of the fiber bundles connecting different ROIs within the macaque brain. The resulting streamlines were visualized using FSLeyes.


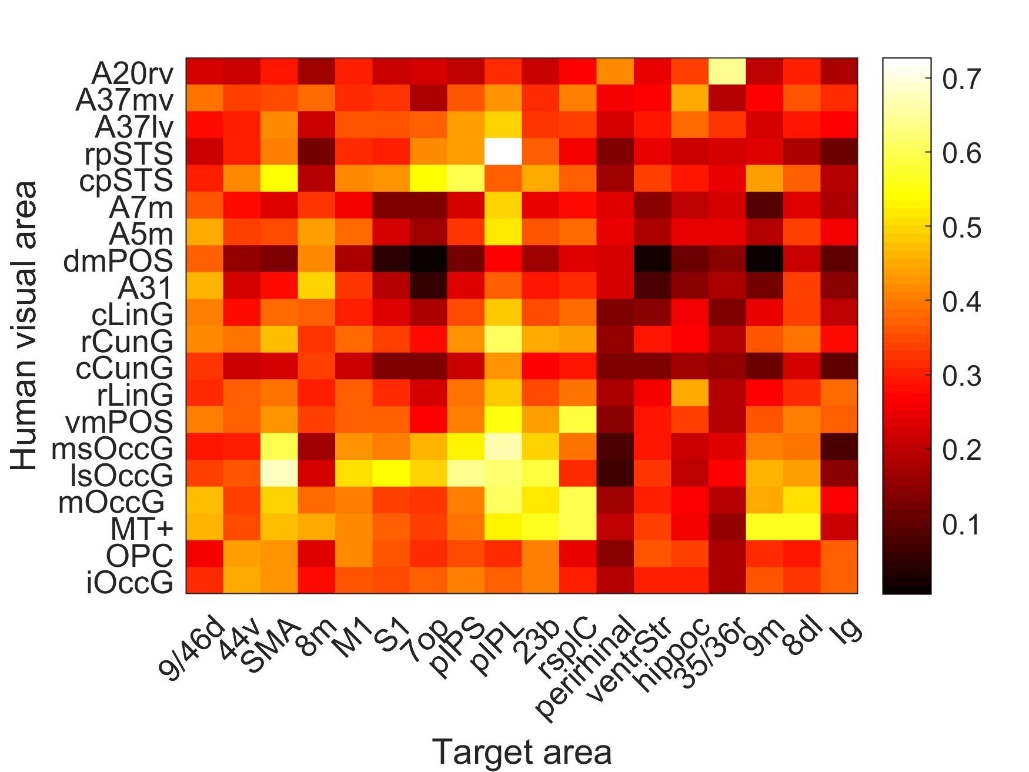


**Supplementary Figure 3.** Functional connectivity results in the human subject. The X-axis represents the homologous target region, the Y-axis represents the ROIs in the human visual system. The values in the middle indicate the correlation coefficients between the ROIs and the target region. The correlation coefficients were defined as the connection strength values after Fisher Z-transformation.


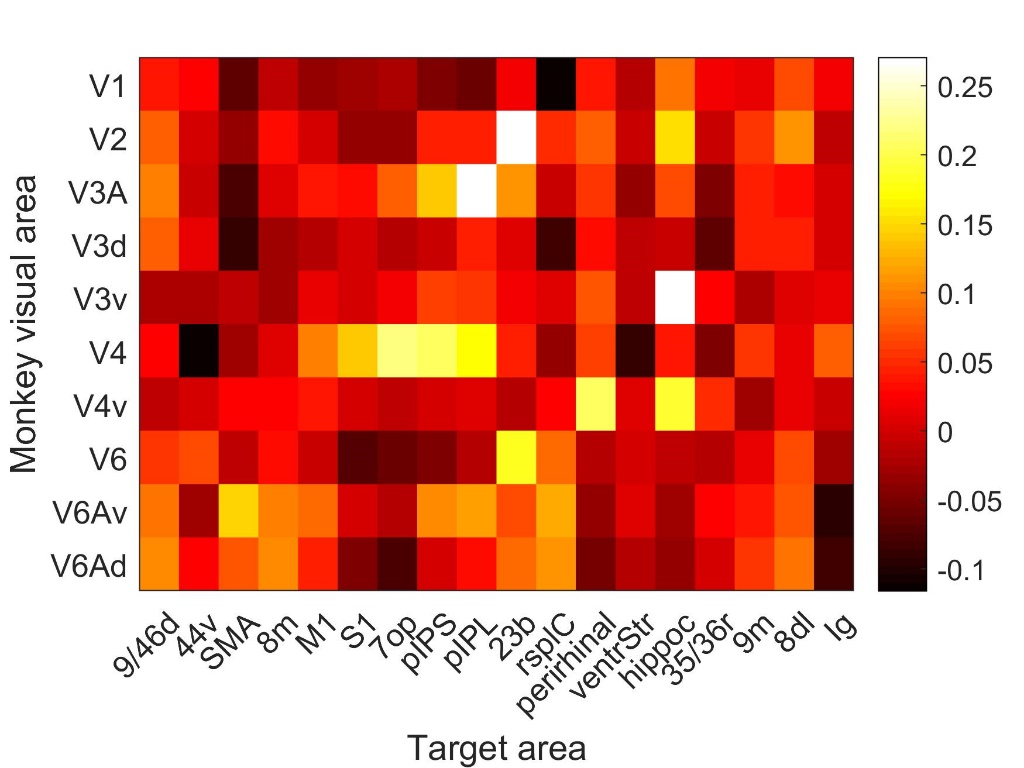


**Supplementary Figure 4.** Functional connectivity results in the macaque subject. The X-axis represents the homologous target region, the Y-axis represents the ROIs in the monkey visual system. The values in the middle indicate the correlation coefficients between the ROIs and the target region. The correlation coefficients were defined as the connection strength values after Fisher Z-transformation.


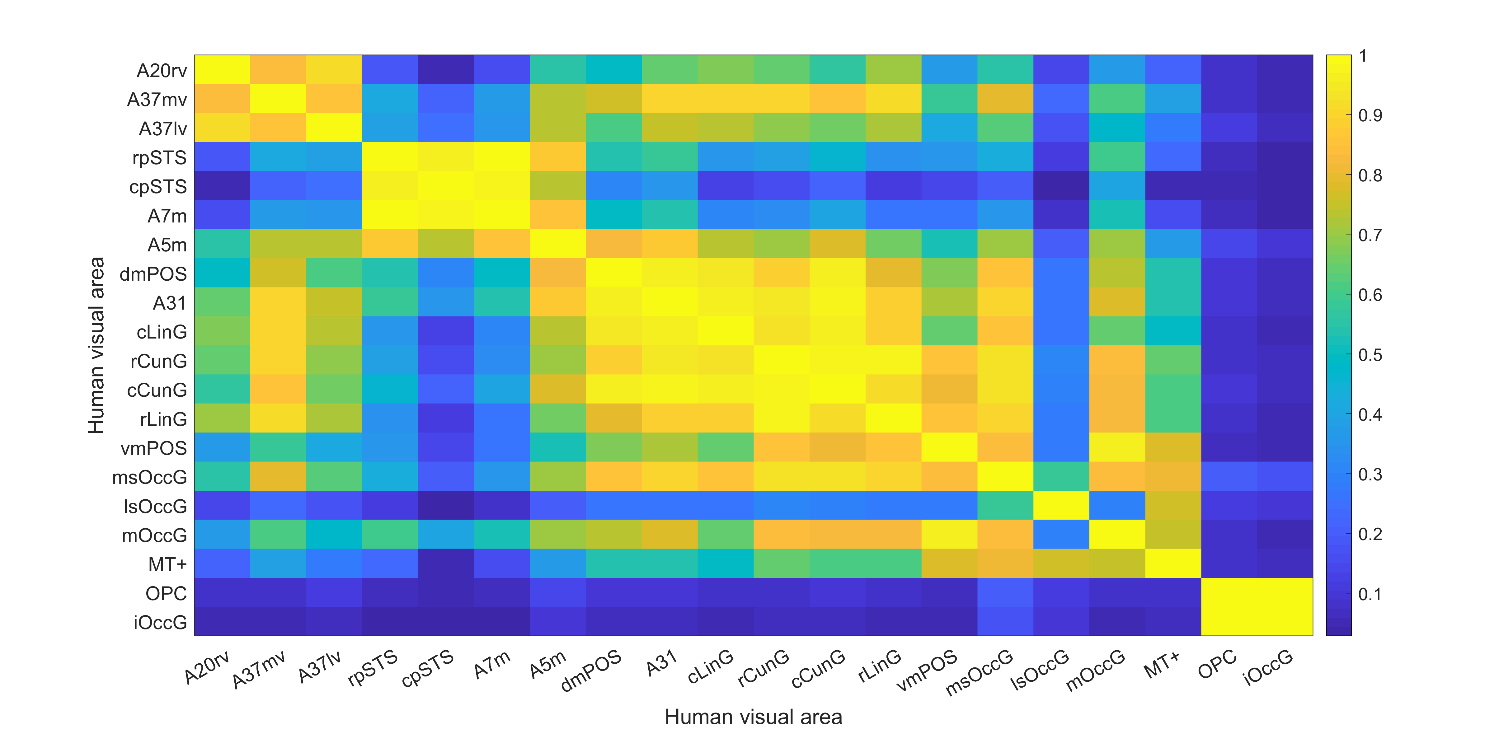


**Supplementary Figure 5.** Intra-species similarity of structural connectivity in the human visual system. The X-axis and the Y-axis represent brain regions in the human visual system. Cosine similarity values are plotted in matrix form. From the figure, it can be observed that the matrix is a skew-symmetric matrix. Comparing the SC fingerprints between the 20 brain regions of the human visual system, it is shown that 83/190 (excluding the diagonal) pairs of regions have cosine similarity coefficients higher than 0.6, indicating that the SC fingerprints among different brain regions of the human visual system are similar. This suggests a high intra-species similarity and consistency in the human brain.


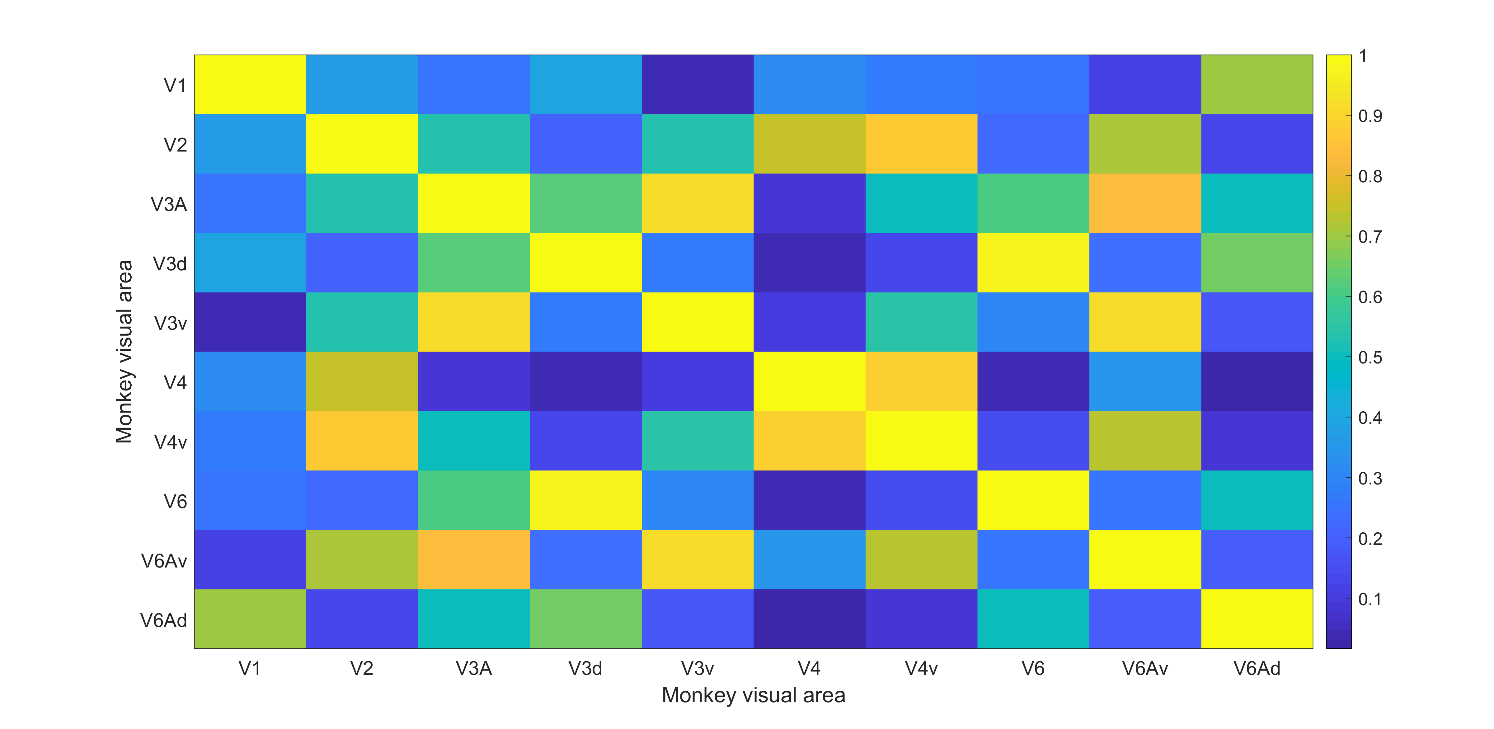


**Supplementary Figure 6.** Intra-species similarity of structural connectivity in the monkey visual system. The X-axis and the Y-axis represent brain regions in the monkey visual system. Cosine similarity values are plotted in matrix form. From the figure, it can be observed that the matrix is a skew-symmetric matrix. Comparing the SC fingerprints between the 20 brain regions of the monkey visual system, it is shown that 13/45 (excluding the diagonal) pairs of regions have cosine similarity coefficients higher than 0.6, indicating that the SC fingerprints among different brain regions of the monkey visual system are similar. This suggests a high intra-species similarity and consistency in the monkey brain.
